# Bibliometric and visual analysis of nocturnal enuresis from 1982 to 2022

**DOI:** 10.3389/fped.2022.972751

**Published:** 2022-08-12

**Authors:** Wenjie Li, Guang Yang, Wenxiu Tian, Yunqi Li, Lei Zhang, Youjie Wang, Yanlong Hong

**Affiliations:** ^1^Shanghai Innovation Center of TCM Health Service, Shanghai University of Traditional Chinese Medicine, Shanghai, China; ^2^Engineering Research Center of Modern Preparation Technology of Traditional Chinese Medicine of Ministry of Education, Shanghai University of Traditional Chinese Medicine, Shanghai, China

**Keywords:** nocturnal enuresis, bibliometric, CiteSpace, pathogenesis, standardization of terminology

## Abstract

Nocturnal enuresis is a common disorder among children that seriously affects physical and mental health and has become a social problem. Bibliometric analysis is a valid way to examine existing research results, current research hotspots and research frontiers. Current studies on nocturnal enuresis are numerous and complex, but a bibliometric analysis of the existing research on nocturnal enuresis has yet to be published. To better identify the research trends and frontiers in nocturnal enuresis, it is necessary to conduct a comprehensive review and analysis. We used bibliometric and visualization methods to analyze the 1,111 papers published between 1982 and 2022 from the Web of Science core collection. Basic information about the country, institution, and authors was analyzed, which led to a basic understanding of nocturnal enuresis. The United States is the most prolific country, Ghent University is the most influential institution, and Rittig Soren is the most prominent scholar. The frequency of keywords, clustering, and the cited literature were analyzed to understand the hotspots and frontiers of research, and a brief review of the highly cited literature was conducted. The current research hotspots are the treatment modalities for nocturnal enuresis, epidemiological investigations, and the exploration of pathogenesis. Clinical research, adenoidectomy, aquaporin 2, and response inhibition are potential research hotspots. The standardization of terminology in nocturnal enuresis and the pathologies of polyuria and sleep disorder are at the forefront of research. In summary, the results of our bibliometric analysis reveal views on the current situation and the trend of nocturnal enuresis research for the first time. This study may provide guidance for promoting research on nocturnal enuresis.

## Introduction

Nocturnal enuresis (NE) is defined by the International Children's Continence Society (ICCS) as involuntary urination during sleep in children aged 5 years or more (1). Based on the cause of the disease, NE can be classified as primary nocturnal enuresis (PNE) or secondary nocturnal enuresis (SNE); furthermore, based on the presence or absence of comorbidities, NE can be classified as monosymptomatic nocturnal enuresis (MNE) or non-monosymptomatic nocturnal enuresis (NMNE) (2). Epidemiological studies have shown that the prevalence of NE varies across regions, but the global overall prevalence ranges from 4 to 16% (3, 4). Nearly 15% of children with mild symptoms recover on their own each year without any intervention (5), but 1–2% of children with severe conditions will continue to experience symptoms into adulthood (6). Moreover, severe NE can lead to troublesome problems such as psychosocial issues, low self-esteem, fear of sleepovers, and social isolation (7, 8); it is also difficult for the families affected.

NE is a complicated disease with multiple pathogenetic factors that have been extensively studied in recent decades. The common symptoms are excessive nocturnal urine production and bladder dysfunction (9, 10). There are other causes, such as sleep-wake disorder, genetic inheritance (11, 12), hormonal dysregulation (13, 14), neurological abnormalities (15), upper airway obstruction, the circadian rhythm of glomerular filtration rate disorder (16), sleep-disordered breathing (17) and so on. However, these mechanisms can only explain some specific phenomena. Research on NE therapies is also diverse. 1) The arginine vasopressin (AVP)-specific analog desmopressin has been applied based on the physiological characteristics of the antidiuretic action of AVP. Anticholinergics, tricyclic drugs, and prostaglandin synthesis inhibitors have also been used. 2) Behavioral therapy includes arousal training, urine holding training, and toilet training (18, 19). 3) Non-invasive therapy includes alarm therapy (20) and electrical stimulation therapy (21, 22). 4) Tonsillectomy is also a treatment option (23, 24). In addition, many researchers have investigated the use of combination therapies (25–28), the underlying mechanisms of the disease (29–31), and the prognosis and adverse reactions associated with these therapies to improve their efficacy and safety (30, 32, 33). However, although there has been some theoretical basis in the pathogenesis, there is no consensus. Second, although many studies have examined treatments for NE, their clinical application still needs to be further explored. Therefore, it is very important to understand the general situation of NE research and identify the trends of NE research.

Bibliometric analysis is a widely applied quantitative method for investigating or reviewing literature in a unique field (34). More information, such as journals, authors, keywords, countries, institutions, references, and other detailed data, can be obtained in this process, and some visual networks can be formed based on those data (35, 36). Analyzing these visual networks makes it possible to understand the direction of the discipline, publication trends, and author citation relationships, among other things. Thus, despite the appearance of diversity of NE publications and research topics, bibliometric and visual analysis can help us quickly understand the field from massive literature information and identify the hot spots of research and the direction of disciplinary development in each period. Additionally, we can also condense and summarize the current results and provide a practical, comprehensive, and informative overview of references for future research in NE.

## Materials and methods

### Data retrieval and data collection

This cross-sectional study retrieved literature information from the Web of Science core collection (WOSCC) (https://www.webofscience.com), which is regarded as the independent global citation database for the most trusted publishers in the world, on April 10, 2022. All searches were conducted on the same day to avoid bias due to daily database updates. The edition we selected is Science Citation Index Expanded (SCI-EXPANDED). The retrieval strategy selected was Topic= (“Nocturnal enuresis” OR “Nocturnal bedwetting” OR “Enuresis” OR “Bedwetting”). Other inclusion criteria are shown below.

(1) Timespan: January 1, 1982, to April 10, 2022.
(2) Language: English.
(3) Document type: Article and Review article.

The search yielded 2,610 articles, and after screening the titles, abstracts, and contents of each article, studies that included retractions, duplications, or irrelevant research were removed. The types of literature not related to NE mainly include the following categories.

(1) Nocturnal enuresis in infant (age < 5).
(2) Nocturnal enuresis in adult (age > 18).
(3) Only daytime urinary incontinence.
(4) Studies that only mentioned “NE” in the article but did not examine this disease.

**Figure 1:**
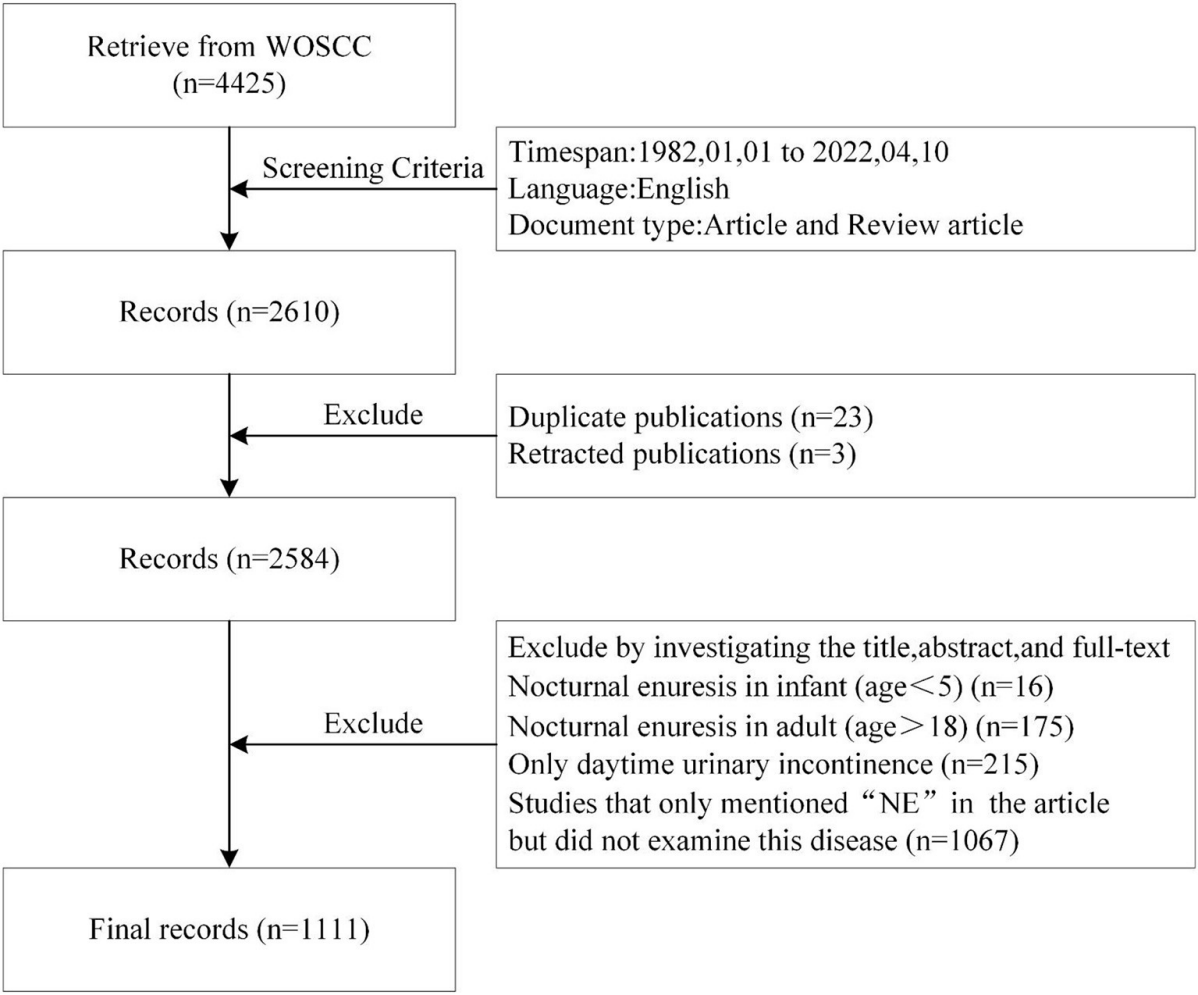
Flowchart depicting the article selection process.

Disagreements in the screening process were resolved through consultation between the authors. Ultimately, 1,111 articles were included in the bibliometric analysis. Figure 1 illustrates the literature screening process. The data with “Full record and Cited references” were exported from WOSCC in “Plain text file”, “Excel” and “Tab-delimited file” formats.

### Data analysis and network mapping

The bibliometric and visual analysis of this work was mainly completed with CiteSpace (Version 5.8. R3, USA), VOSviewer (Version, 1.6.18, USA), Scimago Graphica (USA), and R software (Version 4.2.0, USA) as well as https://bibliometric.com/app.

We separately uploaded the “Tab-delimited file” data to the online website for the analysis of the total publication trends per year and the publication trends per year for different countries and to VOSviewer to generate the country's cooperation network, modified by Scimago Graphica. The remaining analyses, including institutions, authors' collaboration networks, co-occurrence analysis of subjects and keywords, and co-citation analysis of the literature, were completed through CiteSpace using “Plain text file” data. Except where noted, the parameters for CiteSpace settings were as follows: time slicing from January 1982 to April 2022; 1 year per slice. For the Links Strength, we chose “Cosine;” for Links Scope, we chose “Within Slices.” A modified g index was used in each slice: ***g***^2^ ≤ ***k***∑_*i*≤*g*_***c***_**i**_, ***k***∈***Z***^+^, and the scale factor ***k*** = ***25*** (37). In the Pruning window, we choose “Pathfinder,” “Pruning sliced networks,” and “Pruning the merged network” to obtain a more streamlined network for focus, and we did not change any other details. The Node Type was dependent on the project being analyzed. For example, when analyzing the cooperation network with authors, the node of “Authors” must be chosen. In addition, we used the R package “bibliometrix” developed by Massimo Aria and Corrado Cuccurullo (38) to analyze the relationship of authors with their institutions and published journals and calculated the H-index. The generated data were collected in Excel 2021.

## Results

### Annual publication trend

**Figure 2:**
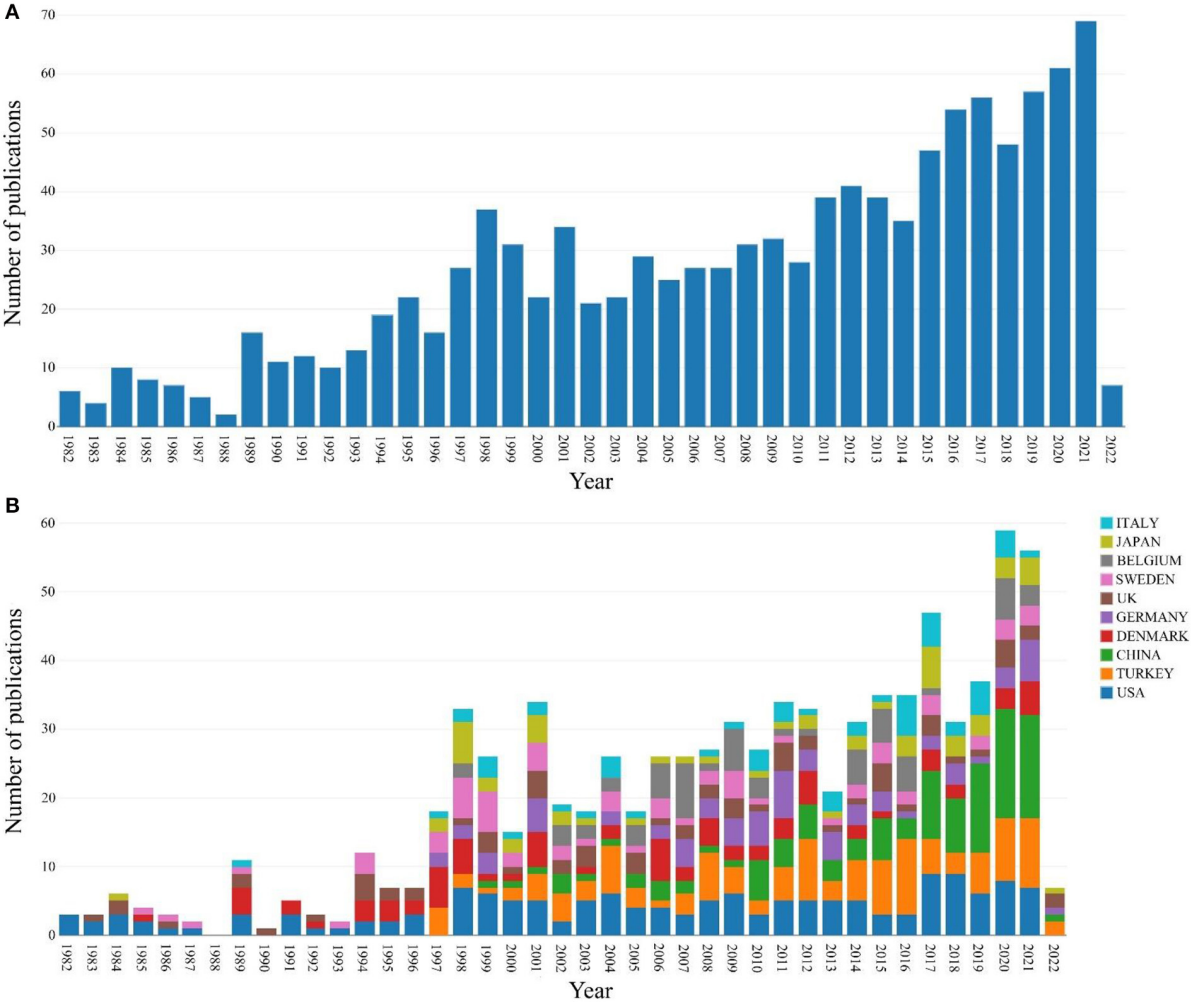
**(A)** The number of annual publications on NE from 1982 to 2022. **(B)** The number of publications per year on NE in different countries from 1982 to 2022.

There were 893 articles (80.37%), 3 early access articles (0.27%), 103 proceedings papers (9.27%), and 112 review articles (10.08%). First, we counted the number of annual publications on nocturnal enuresis from 1982 to 2022. As shown in Figure 2A, the overall publication volume still showed an increasing trend year by year. However, the growth of 1997–2001 showed a short period of rapid rise and decline. Based on this phenomenon, we examined the types of papers issued during 1997–1999, mostly proceedings papers. Furthermore, the proportion of proceedings papers to the total number of publications per year was 1997 (46.15%), 1998 (54.05%), and 1999 (41.93%). Therefore, we speculated that this is the reason for the significant increase in annual publications during 1997–1999. Although, there is a large difference in the number of publications between 2000 and 2001, the proportion of proceedings papers is not the cause of this phenomenon; it is 0% in 2000 and 0.29% in 2001, which may be due to the publication cycle.

We also calculated statistics on the number of published papers in different countries each year, as shown in Figure 2B. The top 10 most productive countries or regions are presented in Table 1. The major evaluation indices include the number of documents, the first publicized year, and citations. Whether it is the total number of papers published between 1982 and 2022 or the trend of publishing papers each year, the top three countries are the United States, Turkey, and China. According to recent annual publishing trends, especially after 2017, the number of publications in China is rising, and China has surpassed the U.S. in the number of articles published each year. However, in terms of the year of research initiation (1982), publications (159), and citations (4,195), the US is the dominant country for NE research.

**Table 1:** The top 10 countries or regions on publications on NE from 1982 to 2022.

**Rank**	**Papers**	**First publicized year**	**Citations**	**Countries or regions**
1	159	1982	4,195	United States
2	124	1997	1,236	Turkey
3	110	1999	1,319	China
4	77	1985	1,165	Denmark
5	70	1983	981	United Kingdom
6	69	1997	1,040	Germany
7	68	1985	1,810	Sweden
8	62	1998	1,355	Belgium
9	56	1984	798	Japan
10	54	1989	1,035	Italy

### Analysis of published journals

An analysis of publications related to NE published in 282 journals revealed that the articles published in the top 10 journals accounted for 45.81%. Table 2 shows the publication volume, citations, impact factors in 2021, JCR partition (https://jcr.clarivate.com/), and publishers. Based on both publications and citations, the Journal of Urology ranks first, with 125 publications and 1981 citations. Thus, many novel and cutting-edge research articles may be found in this journal. Although the Journal of Pediatric Urology and Scandinavian Journal of Urology also have more publications, the former has fewer citations. The impact factor is a quantitative indicator used to evaluate the importance of absolute or total citation frequency. The impact factors of 2021 range from 1.921 to 24.267, with an average of 5.6295. European Urology had the highest impact factor (24.267), demonstrating its dominance in the field of NE. Most of the JCR divisions of these journals are in Q1/Q2, which also indicates that they have potential growth potential. In addition, 80% of the top 10 journals are published by Elsevier and Wiley.

**Table 2:** The top 10 Journals of publications volume on NE From 1982 to 2022.

**Rank**	**Journals**	**Papers**	**Citations**	**IF (2021)**	**JCR**	**Publisher**
1	J Urology	125	1,981	7.600	Q1	Elsevier
2	J Pediatr Urol	94	369	1.921	Q3/Q4	Elsevier
3	Scand J Urol	81	895	1.899	Q4	Informa healthcare
4	BJU Int	46	773	5.969	Q1	Wiley
5	Acta Paediatr	39	595	4.056	Q1	Wiley
6	Pediatr Nephrol	31	277	3.654	Q2/Q2	Springer
7	Urology	29	222	2.633	Q3	Elsevier
8	Neurourol Urodynam	24	86	2.367	Q3	Wiley
9	Eur Urol	21	307	24.267	Q1	Elsevier
10	J Paediatr Child H	17	121	1.929	Q3	Wiley

### Analysis of the cooperation network

#### Countries

**Figure 3:**
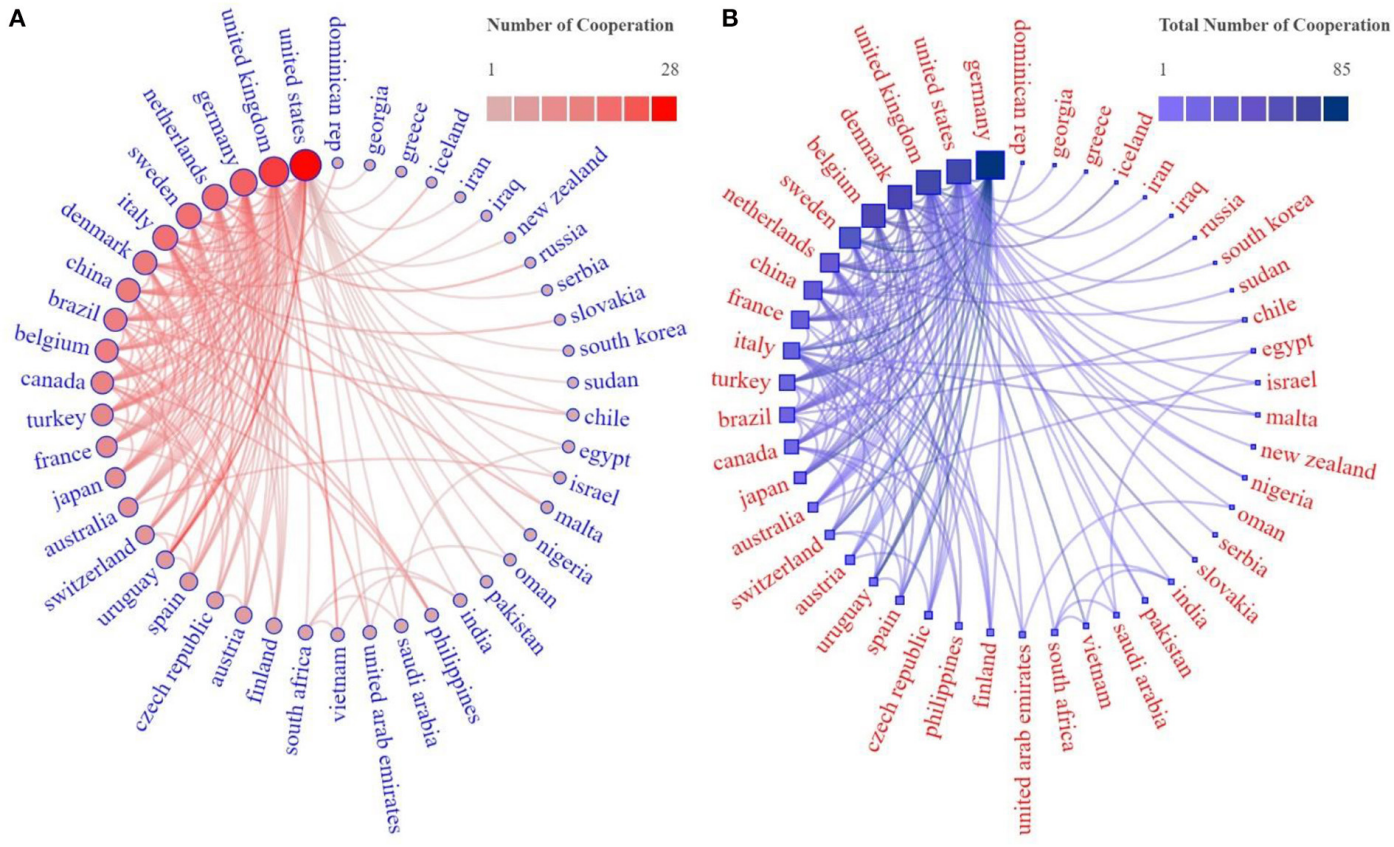
Network of countries' cooperation on NE. Each circle or square represents a country or region, the size of the area and the shade of the color inside represent the frequency of collaborations, and the lines represent collaborative relationships. **(A)** Numbers of cooperation with other countries. **(B)** Total times of cooperation.

Cooperation, interaction, complementarity, and construction between different countries or institutions have far-reaching significance, and the analysis of cooperation relations can identify influential countries and institutions. Thus, we combined two tools, VOSviewer and Scimago Graphica, to visualize and analyze the cooperation relationships between countries or regions. Countries not involved in cooperation were excluded. The results are shown in Figure 3. Forty-six out of 61 countries engaged in international cooperation. For NE research, the U.S. had the most collaborators, reaching 28, followed by the U.K. and Germany (Figure 3A). However, Germany seems to show more activism in terms of cooperation, with 85 collaborations with other countries. The U.S. is tied with the U.K. for second with 63, and Denmark is in third place (Figure 3B), suggesting that cooperation and communication with these countries can be strengthened with respect to researching NE.

#### Institutions

Regarding institution-to-institution collaboration, 1,266 institutions participated in the NE study, and Table 3 lists the top 10 institutions in terms of total publications and includes citations and country of residence. Supplementary Table 1 shows the total number of papers published by these institutions as first completers and the number of citations and the average number of citations. It can be seen that Ghent University has the highest number of publications (100, 1,238 citations), while Aarhus University (93, 1,355 citations) and Uppsala University, Sweden (57, 1,038 citations) follow closely behind (Table 3). Additionally, these three institutions are also in the top three according to the number of first-author publications (Supplementary Table 1), showing that these three institutions play a dominant role in the NE field. Interestingly, 1/3 of the institutions are from China, in which China Medical University has more publications (39) (Table 3), and the Chinese University of Hong Kong has the highest average citation rate (21.62) (Supplementary Table 1). Regarding the distribution characteristics of the institutions, Denmark and New Zealand have a small and relatively concentrated group of research institutions. In contrast, the absence of US and Turkish research institutions on the list suggests that their research institutions may be numerous and dispersed.

**Table 3:** The top 10 institutions of publications volume on NE from 1982 to 2022.

**Rank**	**Institutions**	**Papers**	**Citations**	**Countries**
1	Ghent University	100	1,238	Belgium
2	Aarhus University	93	1,335	Denmark
3	Uppsala University	57	1,038	Sweden
4	China Medical University	43	244	China
5	São Paulo University	31	120	Brazil
6	Shanghai Jiao Tong University	28	146	China
7	Saarland University	25	229	Germany
8	Sydney University	25	273	Australia
9	Chinese University of Hong Kong	23	513	China
10	Children's Hospital Westmead	20	151	Australia
11	Zhengzhou University	20	80	China
12	Juntendo University	17	42	Japan

**Figure 4:**
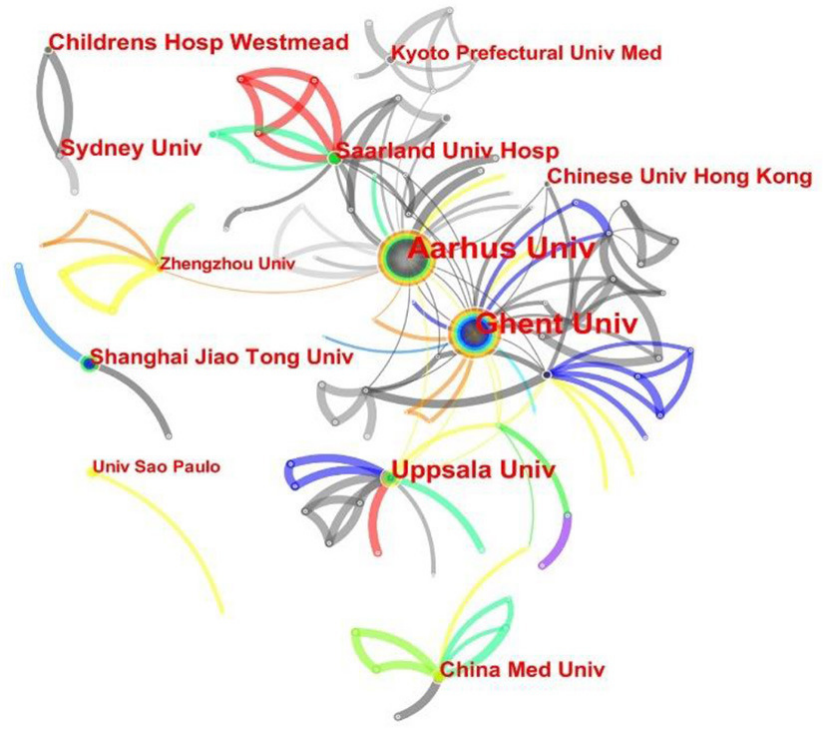
Network of institutions' cooperation on NE research. Each circle represents an institution. The size of the circle is positively related to the number of articles published by the organization, and the thickness of the line is positively correlated with the frequency of cooperation.

The results of the analysis of CiteSpace are shown in Figure 4. Although there is a collaboration between institutions with a high volume of publications, there is a tendency to collaborate with other institutions that possess fewer publications. Children's Hospital Westmead and Sydney University seem to collaborate closely, likely because they are both located in Australia. In contrast, China has four institutions on the list, namely, the Chinese University of Hong Kong, China Medical University, Shanghai Jiao Tong University, and Zhengzhou University, but they have never seemed to collaborate.

#### Authors

By analyzing the number of articles published by the authors and the collaboration network, we found that 3,638 authors researched NE. Additionally, we can see from the data of the top 10 authors in Table 4 that the bulk of them are from Belgium and Denmark. Supplementary Figure 1 shows the organization to which the top 10 authors belong and periodicals' publications. Topping the list is Professor Rittig Soren from Aarhus University, who also chairs the scientific committee of the International Children's Continence Society, with 56 papers and 1,118 citations. His major research fields are water balance, pediatric nephrology, kidney, enuresis, renal disease, nephrotic syndrome, and gene expression. The second-ranking author is Professor Vande Walle Johan, from Ghent University, with 46 papers and 829 citations. His main research fields are pediatric drug research, enuresis, nycturia, uremic toxins, hyperoxaluria, and COVID in children. Moreover, the H-index is often used to evaluate the quantities and levels of academic output of researchers. The authors in the top 5 have a high H-index exceeding 20, which indicates that they have a considerable influence on NE research.

**Table 4:** The top 10 authors of publications volume on NE from 1982 to 2022.

**Rank**	**Authors**	**Papers**	**Citations**	**H-index**	**Affiliations**	**Countries**
1	Rittig S	56	1,118	23	Aarhus Univ	Denmark
2	Vande Walle J	46	829	23	Ghent Univ	Belgium
3	Hoebeke P	36	464	21	Ghent Univ	Belgium
4	Djurhuus JC	35	679	19	Aarhus Univ	Denmark
5	Von Gontard A	34	521	16	Saarland Univ	Germany
6	Neveus T	33	569	18	Uppsala Univ	Sweden
7	Kamperis K	21	170	12	Aarhus Univ	Denmark
8	Raes A	19	207	11	Ghent Univ	Belgium
9	Eggert P	18	280	10	Kiel Univer	Germany
10	Van Herzeele C	15	146	10	Ghent Univ	Belgium
11	Caldwell PHY	15	138	8	Univ of Sydney	Australia

**Figure 5:**
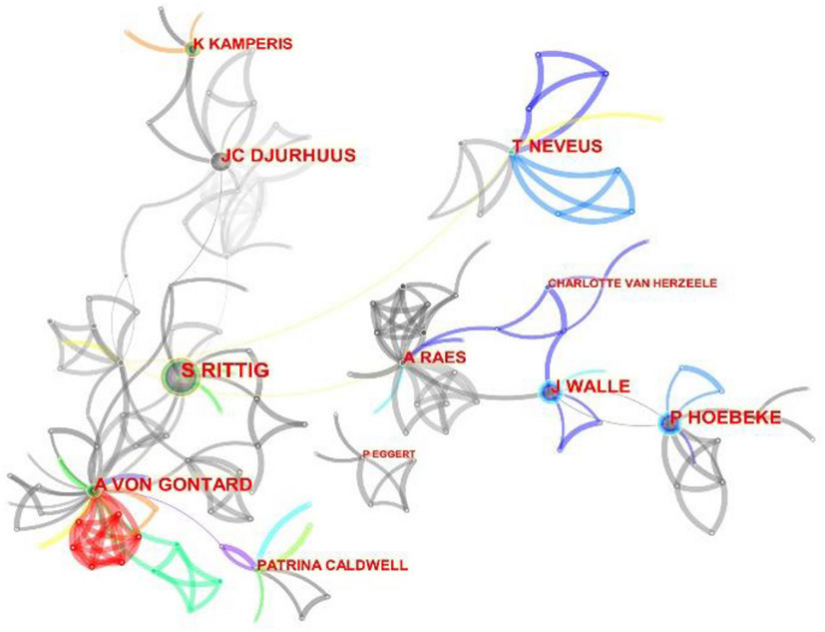
Network of authors' cooperation on NE research. Each circle represents an author. The size of the circle is positively related to the number of articles published by the author, and the thickness of the line is positively correlated with the frequency of cooperation.

By analyzing their cooperative relations, we find that most scholars have established cooperative relations, and their cooperative relations are intertwined to form a centralized group (Figure 5). The cooperative relations among Hoebeke Piet, Vande Walle Johan, and Raes Ann are relatively close. Djurhuus Jens Christian and Kamperis Konstantinos also have a close cooperative relationship. Rittig Soren and von Gontard Alexander have established a close cooperative relationship. In comparison, Neveus Tryggve and Eggert Paul prefer to cooperate with other authors that have a small number of articles.

### Analysis of the co-occurrence network

#### Interdisciplinary

To explore the combination and development between disciplines in NE, the analysis of subject co-occurrence was used, and the associated network was established. As displayed in Supplementary Figure 2, NE research involves multiple disciplines due to its complex pathogenesis and multiple treatment methods. Urology & nephrology and pediatrics are the two main subjects categorized by the pathological features of NE, mainly manifested in abnormal urine metabolism among children. Medicine, general & internal, and pharmacology & pharmacy are also important subject categories, including randomized, double-blind clinical trials, pharmacodynamics, *in vivo* mechanisms of action, and safety and toxicity tests for NE. In addition, the role of neurosciences and neurology, psychiatry, psychology, otorhinolaryngology, dentistry, oral surgery and medicine in exploring the pathogenesis and developing new treatments cannot be ignored.

#### Keywords

**Figure 6:**
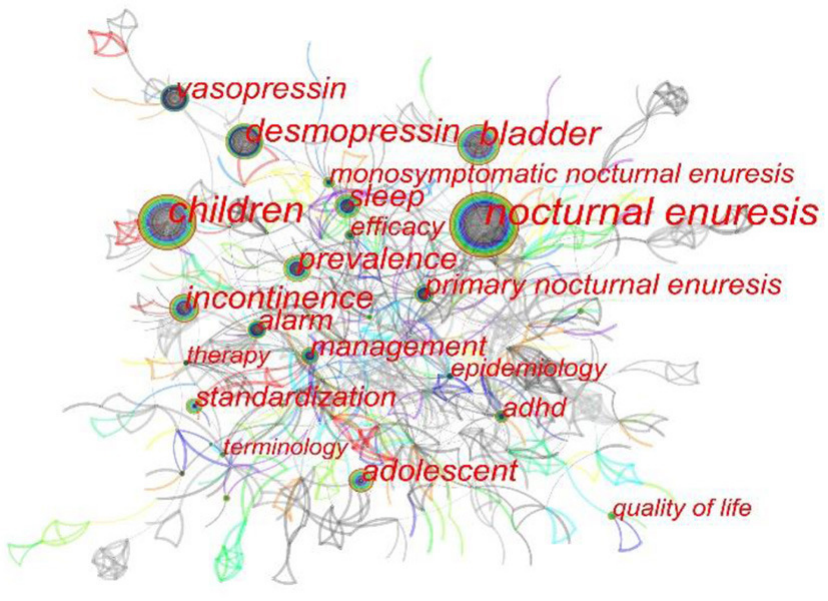
Network of keywords occurrence on NE research. Each circle represents a keyword. The size of the circle is positively related to the frequency of the keyword, and the thickness of the line is positively correlated with the degree of association.

Keywords are used to express the document's subject matter, and the research field of hotspots and directions can be reflected in keyword co-occurrence. Figure 6 shows the correlation network between keyword occurrence. The ligature between nodes is sophisticated, which shows that the connection between keywords is intimate and that the cooperation in different fields is comprehensive. We used CiteSpace to extract the keywords and incorporated some with the same meaning but different spellings. Supplementary Table 2 shows the top 20 popular keywords. Among these keywords, “nocturnal enuresis,” “children” and “adolescent” represent the pathological features of NE. “Primary nocturnal enuresis” and “monosymptomatic nocturnal enuresis” are two different subtypes of NE. “Desmopressin” and “alarm” indicate the mainstream therapeutic approaches. Functional disorders related to “bladder,” “vasopressin” and “sleep” are potential pathogenic mechanisms. Attention deficit and hyperactivity disorder (“ADHD”) is the most common complication of NE. In addition, “prevalence,” “epidemiology,” “standardization,” “terminology” and “quality of life” appear more frequently and are widely used keywords in the field of NE.

**Figure 7:**
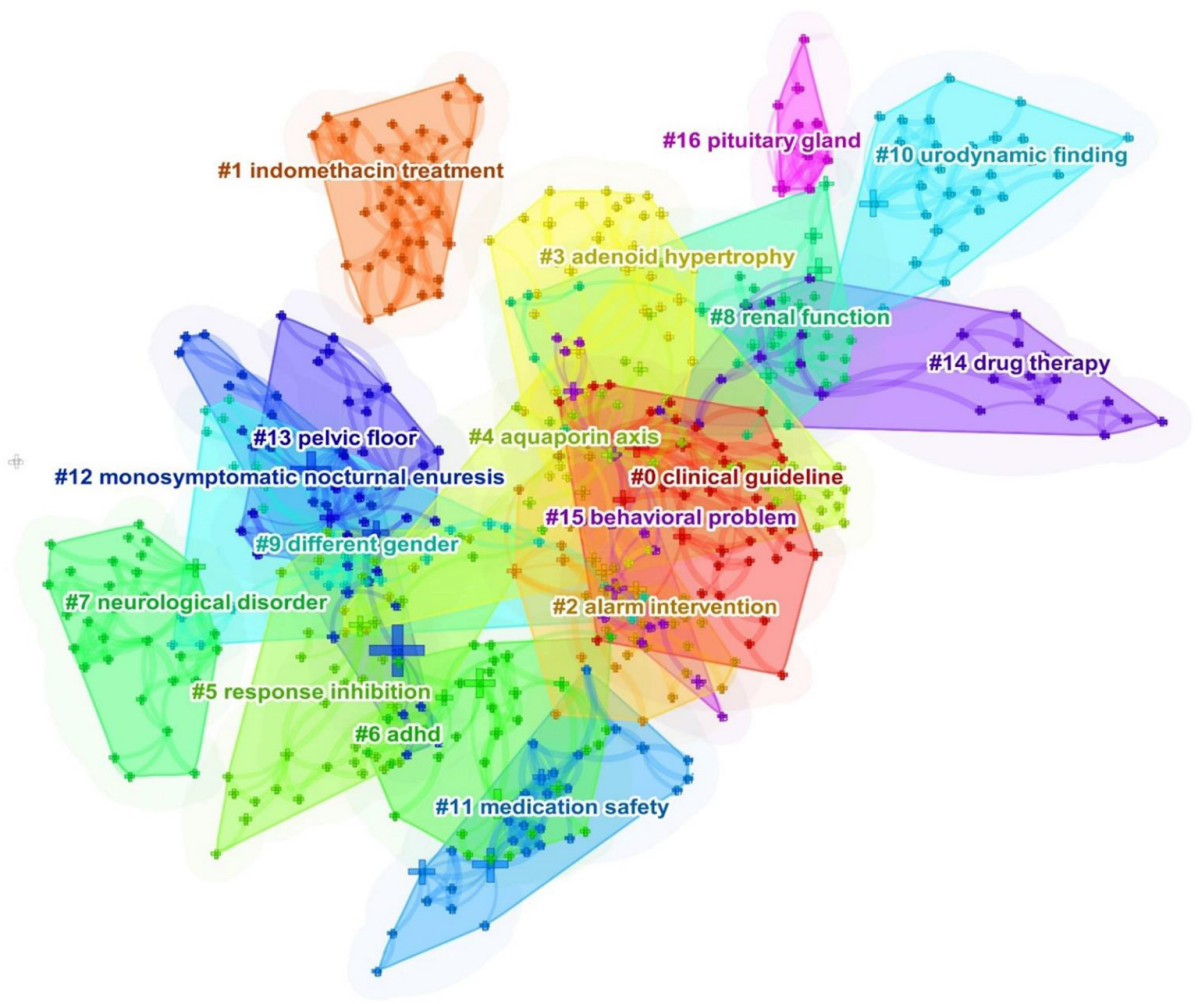
Clustered networks of keywords. Each color represents a cluster. The smaller the clustering label, the more articles in the cluster (Q = 0.765, S = 0.905).

To derive the basic status of each research topic within the NE research area. We also analyzed the cooccurrence keyword clustering and used CiteSpace to build the visual network map (Figure 7). Clustering tags are significant noun phrases extracted from the article titles. Co-occurrence keywords can be divided into 17 groups: #0 clinical guideline, #1 indomethacin treatment, #2 alarm intervention, #3 adenoid hypertrophy, #4 aquaporin axis, #5 response inhibition, #6 ADHD, #7 neurological disorder, #8 renal function#, 9 different genders#, 10 urodynamic finding, #11 medication safety, #12 monosymptomatic nocturnal enuresis, #13 pelvic floor, #14 drug therapy, #15 behavioral problem, and #16 pituitary gland. The clustering results can be measured by the silhouette value (S) and modularity Q value (Q). The higher the two values are, the better the clustering results obtained by the network. When the S value is > 0.5, the clustering result is considered reasonable, while when it is > 0.7, the result is considered to be highly reliable. When the Q value is > 0.5, it indicates that the network clustering structure is significant (36, 40).

**Figure 8:**
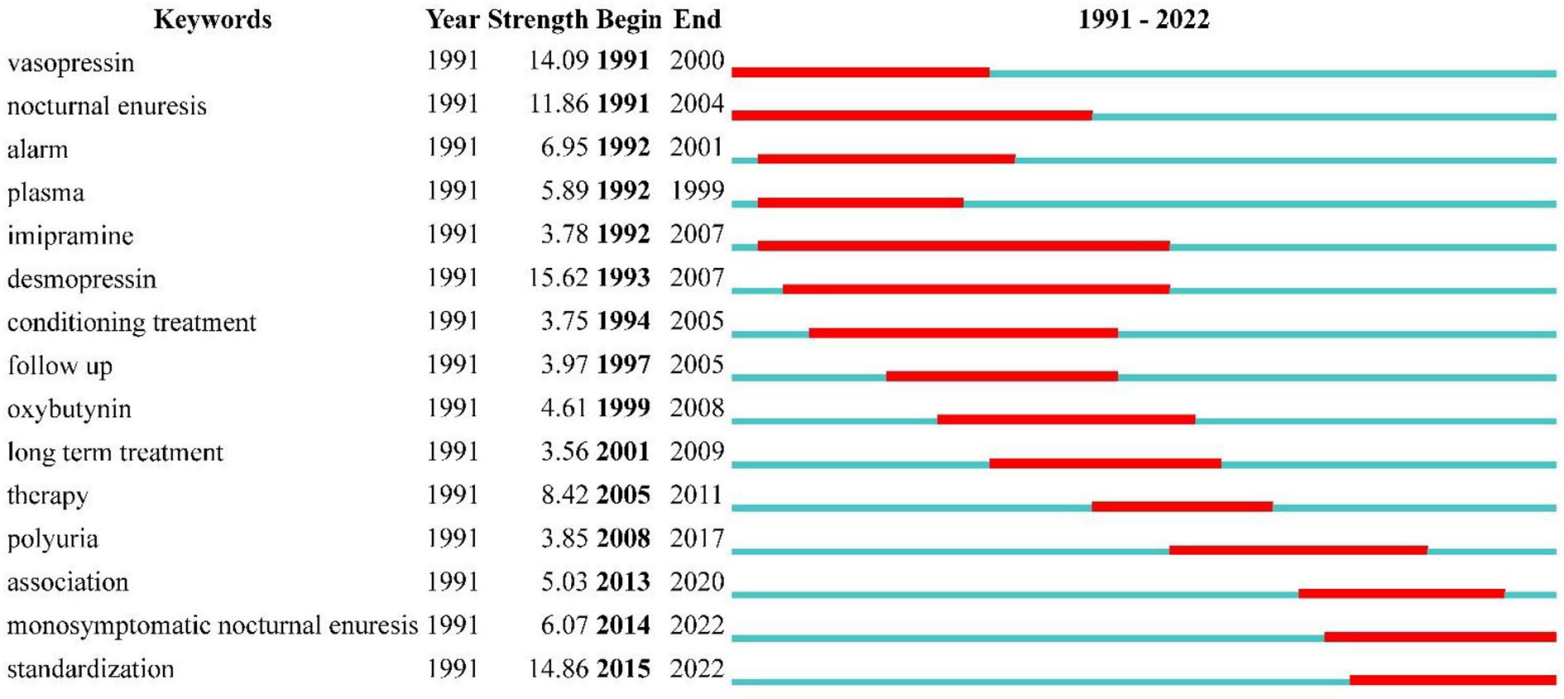
Top 15 Keywords with the Strongest Citation Bursts. The keyword marked in red indicates a sudden increase in the frequency of the keyword during this period. Keywords marked in blue indicate a period of relative unpopularity.

Additionally, we also performed keyword burst detection, keywords that have been cited multiple times over a certain period and are often considered indicators of cutting-edge topics. Since burst keywords were produced only from 1991 onward, we set it as the initial analysis year. The results are shown in Figure 8, where desmopressin, standardization, and vasopressin bursts are high in intensity and lengthy in duration. The initial outburst keywords generated were vasopressin and nocturnal enuresis. The year 1992 generated the most outburst keywords: alarm, plasma, and imipramine. New outburst keywords appeared every year or years thereafter. Moreover, after 2014, more focus was placed on research on monosymptomatic nocturnal enuresis and standardization of terminology.

### Analysis of the reference co-citation network

**Figure 9:**
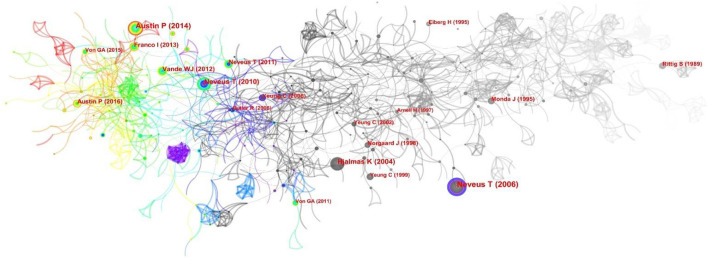
Network of co-cited references on NE. Each circle represents a citation, the size of the circle is positively related to the cited frequency, and the link between the two circles represents the two references cited in the same article.

The co-citation of literature as a research method to measure the relationship between documents was first introduced by the American intelligence scientist Small, Henry in 1973 (41). We performed a series of related analyses using CiteSpace. Figure 9 shows the interaction network of these documents. For the citations, we find that the top 8 most cited documents can be divided into two categories: the first is about the standardization of terminology in NE, while the second is about management guidelines. Undeniably, these articles provide firm, clear guidelines on the terminology of nocturnal enuresis, which are the help of clinical diagnosis and treatment or other studies making it easier to compare studies and decrease confusion among researchers. However, we wanted to use the literature co-citation analysis to determine which had a significant impact and landmark research in the NE field. Therefore, we also analyzed the 10 articles with the highest total number of citations. One was a review, and 9 were articles. Supplementary Table 3 lists the title, year and the first author of those cited references. Among the top citations was a large epidemiological study published by Yeung, C. K in BJU International, which found that adolescent subjects had more severe enuresis symptoms than children by comparing differences in the characteristics of nocturnal enuresis in children and adolescents and that spontaneous resolution of PNE occurred only in children with mild symptoms. Finally, he pointed out that the possible reason for the low prevalence of NE in previous epidemiological surveys in Hong Kong was parental indifference (42). In addition, Yeung, C. K has previously published two articles on the function of the bladder in PNE, revealing that various types of bladder dysfunction are critical considerations in pathologies of PNE. The reduction in nocturnal functional bladder capacity was probably the main cause of a mismatch between nocturnal urine output and bladder storage capacity in patients with severe NE that was refractory to treatment (43, 44). The articles with the second-highest citations include two papers. One of them was published in the American Journal of Physiology in 1989. The investigators compared 15 children with enuresis and 11 normal children and showed that patients did not have the same circadian rhythm of AVP as normal children and that nocturnal AVP levels were lower among patients. Thus, abnormal AVP circadian rhythm seems to be an essential pathophysiological factor for NE (45). The other one was published in Journal of Urology in 1995. This clinical trial used different therapies in 261 patients, among which imipramine, desmopressin, and alarm therapy were all influential in PNE. However, only the effect of alarm therapy lasted, indicating that alarm therapy was the most effective therapy (39). Two others are genetically related, including one published in 1995 in Nature Genetics, in which 11 families with a history of PNE were analyzed by genetic recombination techniques, suggesting that causative genes are likely to be located at markers flanking chromosome 13q13-q14.3 (12). A subsequent article published in Journal of Medical Genetics in 1997 conducted a similar study, again demonstrating the association between PNE and the ENUR1 locus at chromosome 13q, in addition to also showing the presence of a second locus of PNE on chromosome 12q (11). Further research is still needed.

**Figure 10:**
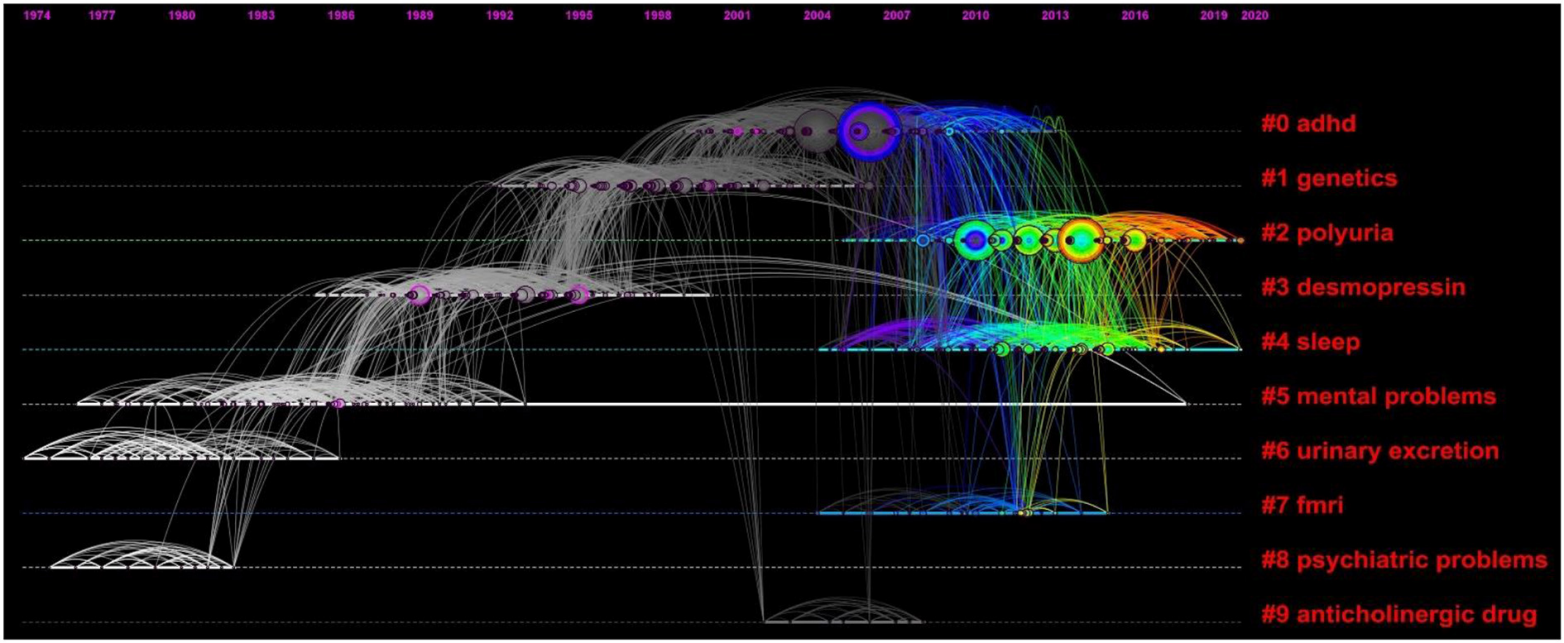
Timeline view of the top 10 largest clusters of citing articles in the field of NE. The highlighted and thickened timeline indicates that the cluster theme is a hotspot during this period (Q = 0.6894, S = 0.8798).

The timeline view reflects the time span over which the literature has been cited. Each row is a collection of similar literature, thus allowing the novelty and continuity of the research topic to be determined. Figure 10 shows the 10 groups of classification of the 1,111 cited papers by keyword clustering, including #0 ADHD, #1 genetics, #2 polyuria, #3 desmopressin, #4 sleep, #5 mental problems, #6 urinary excretion, #7 functional magnetic resonance imaging (fMRI), #8 psychiatric problems, and #9 anticholinergic drugs. The more forward the clustering label is, the more documents that are cited. We found that, in the early days, the main focus of the research was on urinary excretion and psychiatric problems. However, it was later confirmed that psychiatric problems were the result of NE rather than the cause, and thus, they lasted for a short time (7). The use of desmopressin became a research hotspot in the early 1980s until the end of the 20th century. In the 1990s, gene analysis techniques were booming. Thus, gene technology, especially genetic linkage analysis, is often used for genetic studies related to NE (46). Between 1990 and 2010, there was a greater focus on the association of NE with ADHD. Additionally, fMRI technology was gradually used to study NE pathogenesis. Currently, polyuria and sleep seem to be the frontiers of research, which may be related to the mechanisms of NE pathogenesis, namely, bladder dysfunction and sleep-wake disorders. Moreover, the mental problems of children have received continuous attention from the 1970s to the present.

## Discussion

### General information

Although there are previous literature reviews related to NE, to our knowledge, this is the first time that we have combined bibliometric and visualization methods to analyze the NE domain, which exhibited our systematic understanding and viewpoint from the past 40 years. We obtained 1,111 NE-related papers from WOSCC and, through collaboration, co-occurrence, and co-citation analysis, reviewed the overall structure as well as identified hot spots.

During the 40 years covered by this study, the amount of literature related to the field of NE has shown a yearly increase and is still on the rise in the coming years. To a certain extent, the number of national publications and citations is an indication of the level of the research field. Furthermore, the uneven development of research in different countries or regions may be related to people's perceptions and the level of discipline building. NE has long been considered to occur in children as a normal phenomenon in recent years because some patients disappear with age and has received little attention. Notably, other countries, such as Denmark and Iceland, have already established large databases to facilitate support for NE research (47). There is no doubt that most of the influential countries are currently located in the occident, with the United States leading the way as a country with a high volume of published articles and citations. However, based on the trend of publications in recent years, China appears to be doing well and should grasp this trend to conduct deeper, broader, and more influential research.

It has become an international trend for different countries, institutions, and scholars to work together on a particular study, which is also true in the NE field. Thus far, Germany seems to be the most active in cooperation. However, there are only one German affiliates among the top 10 institutions. The top three institutions are Ghent University in Belgium, Aarhus University in Denmark, and Uppsala University in Sweden. In addition, 1/3 of the top 10 institutions are from China, which shows that China also occupies an important position in international cooperation. However, it is essential to note that strengthening domestic cooperation is equally important. In any case, the collaboration will further promote academic progress, communication and industry standardization. The terminology related to NE is updated every few years (1, 2, 48, 49), and this is the result of cooperation among scholars from different countries and institutions.

### Research hotspots

Generally, keyword co-occurrence analysis and keyword clustering can reflect hot research spots. Based on the results of the keyword occurrence frequency analysis, three research priorities in the field of NE were identified: treatment, prevalence, and pathogenesis.

#### Treatment

Desmopressin and alarm therapy are commonly used and are recommended by ICCS as the first-line treatment modality for NE (1). The keyword burst detection displayed that they have been studied since the 1990s, and both lasted for a considerable period. During this time, desmopressin has been studied in-depth regarding dosing modality, dose, pharmacokinetic (PK)/pharmacodynamic (PD) and side effects (50–53). The pharmacological action of desmopressin is to act on V2 receptors in the distal tubules and collecting ducts of the kidney, increasing the tubular reabsorption of fluid and thus having a significant antidiuretic effect (54, 55). However, 20 to 60% of children do not respond to treatment and are resistant to desmopressin (56). It is also accompanied by a strong withdrawal reaction and a high recurrence rate. Thus, research on desmopressin is continuing. While there is no unanimous conclusion on the therapeutic mechanism of alarm therapy, there is no doubt about its effectiveness and safety (57, 58). The enuresis alarm is considered to be the only one with definite therapeutic potential (20, 59). However, the downside is the long treatment period and instability of efficacy, which are time and assumption of risk ordeal for both patients and parents. Nevertheless, they have also been used as a positive treatment group during clinical trials (58, 60, 61). Therefore, they continue to be a research priority in terms of treatment. For other treatments, although they are not reflected in the key words, they should also attract people's attention. Recently, textile underwear with leakage sensors has also been available for the treatment of NE, which has high sensitivity and durability compared to an enuresis alarm and may improve the quality of life of affected children (62). In addition, herbal medicine in the treatment of NE has also been reported. Through randomized, double-blind, placebo-controlled trials, “chamomile oil” (63), “Urox ®” (a capsule of extractive *Crataeva nurvala* bark, *Equisetum arvense* stem and *Lindera aggregate* Sims) (64) and “Suo Quan” (a mixture of *Alpinia oxyphylla Miq*., *Dioscorea opposita Thunb*., and *Radix Lindera*) (65), all of them significantly reduced the frequency of NE and had a low recurrence rate without causing any adverse events (63–66). *Yokukansan* (a preparation of kampo) may also be effective, but clinical trials need to be further expanded (67).

#### Prevalence

Prevalence and epidemiology research are also hotspots and have already been conducted in different countries at different levels and scales (3, 4, 68). These works aim to provide insight for epidemiologists to understand the patterns associated with disease occurrence, which can help inform research on etiology, risk factors, and other associated comorbidities. Second, these studies facilitate the development of targeted and effective prevention programs, the design of more reliable diagnostic modalities and interventions, and the formulation of appropriate health policies for a country. It is worth noting that the discovery of differences in the prevalence of boys and girls previously derived from epidemiology (68) has been applied to study the characteristics of NE and the impact of NE on the mental health of patients (69). However, in a limited number of cases, researchers do not yet have a comprehensive picture of the prevalence of NE and are therefore unable to make judgments and assessments.

#### Pathogenesis

In addition, these potential causes of morbidity, such as bladder, vasopressin, and sleep, are also shown on the top 20 keyword lists. These factors have been widely reported (6, 47). At present, there are two theories about bladder function derangements. One is bladder detrusor hyperactivity, embodying some children with enuresis who can be treated with oral anticholinergic drugs or intravesical injection of botulinum toxin (70) and a great overlap between nocturnal enuresis and urgent urinary incontinence (71, 72). The other is reduced bladder functional capacity (BFC) (73, 74). However, it is not clear whether reduced BFC is due to detrusor instability, uncoordinated urination or dysfunction of the central nervous system micturition center (75). Currently, the reported mechanisms of polyuria are innumerable. However, a more mainstream theory lies in the insufficient secretion of AVP. A circadian rhythm abnormality in AVP secretion has been previously reported in NE patients (45). This may be strong evidence of insufficient secretion of AVP at night. However, the underlying mechanism of the association between AVP and NE has yet to be fully elucidated. Sleep also has far-reaching implications in the pathophysiology of NE, and a review published in Sleep Medicine Reviews in 2020 various detailed aspects of the NE effects of sleep, such as arousal dysfunction and autonomic, hemodynamic, and bladder function (7). However, the extent and nature of sleep's role in NE are still up in the air, and the challenges facing research are serious. However, the search for pathogenesis has never stopped. Thus, this is still a hotspot of research.

#### Keyword clustering

Varieties of labels in the keyword clustering showed an association with NE. According to the results of clustering, it can also be divided into two aspects.

One is the clinical guidelines of NE. Clinical guidelines are important guidance for clinicians in practical work, and their value comes from the experimental evidence obtained by clinical research (76). According to the “GRADE” classification standard, the evidence obtained through randomized controlled trials (RCTs) is considered to be of high quality and classified as level 1 (77). Through these tightly controlled trial conditions and random grouping of subjects, RCTs can significantly reduce trial bias and eliminate the impact of individual differences on clinical trial results as much as possible (78). However, the guidelines are not fixed; rather, they are constantly summarized and updated, thus allowing them to truly play a guiding role. Therefore, the development of high-quality clinical trials may also be the focus of future research. Supplementary Table 4 lists recent randomized controlled trials on NE, which are expected to provide guidance for the updating of clinical guidelines for NE treatment.

Second, clustering also includes the exploration of some disease mechanisms. Previous studies have indicated that the prevalence of NE is positively correlated with the severity of obstructive sleep-disordered breathing (SDB) (79), which could be caused by adenotonsillar hypertrophy. Clinical trials show that the symptoms of NE can be significantly improved after adenoidectomy, and the rate of improvement is dramatically higher than the rate of natural remission (23, 80–82). However, limitations remain evident. With a high risk of bleeding and the possibility of postoperative edema, vomiting, and dysphagia (83), research is still needed. Moreover, aquaporin-2 (AQP2) is a key protein that regulates the water permeability of the kidney collecting duct, playing an important role in the regulation of renal water balance (84). In early research, the diurnal ratio of AQP2 correlates with the severity of NE, and once transport is blocked combined with lower AVP levels, it may lead to NE (85). Based on this property, the potential application of AQP2 in NE has been gradually identified by researchers as a biomarker of desmopressin's therapeutic effects (86). Furthermore, the deficiency of response inhibition of the prefrontal cortex (PFC) is accompanied in patients with PNE (87). Additionally, it is a critical feature of executive dysfunction in people with ADHD (88). A recent genome-wide association analysis showed that there is a genetic overlap between NE and ADHD (47). Showing solicitude for response inhibition may help us further explore the relationship between these two diseases. Although there are few complete reports on the above, only when they are mentioned in the article can they be reflected by clustering. Therefore, they may be potential research hotspots.

### Research frontiers

To some extent, the appearance frequency and burst of standardization of terminology also emphasize that the standardization of terminology about NE is also crucial. Currently, different organizations have different definitions of NE, such as the DSM-5 issued by the American Psychiatric Association (APA), which defines NE as several wet nights (≥1 per month) after the age of 6 years old (89). In contrast, the World Health Organization (WHO) International Classification of Diseases (ICD-10) defines NE as children over 5 years of age who maintain a frequency of at least one episode per month for more than 3 months (90). The definition of ICCS is described earlier. Since different rubrics are used, this will likely lead to difficulties in reaching consensus among researchers in different countries and lies in the difficulty of developing a standard treatment. Second, NE, as one of the lower urinary tract dysfunctions in children, can be easily confused with other conditions, such as incontinence (91). It is likely that the incontinence that frequently appears in the keyword list was confused or did not distinguish between these two concepts in the previous study. In keyword burst detection, “standardization” has been cited since 2014 and has high strength, suggesting that standardization of terminology may be at a frontier of research.

Reference related to “polyuria” and “sleep” have been cited many times in recent years with a continued trend. These keywords are also major factors in NE, which shows that they are both topical and on the cutting edge.

## Conclusions

This paper systematically summarizes and analyzes the contributions of countries, institutions, and researchers in the field of NE using bibliometric methods. NE has attracted the attention of many researchers. Specifically, the physiology and pathology of NE are still unclear, and the treatment methods are relatively concentrated. Thus, more research is needed on these aspects. However, studies need to be based on a consensus among researchers. In general, the research in this article is based on the published literature, and we hope to provide a valid reference for the development of NE.

## Limitations

First, we searched only relevant literature in WOSCC. Although it is the most comprehensive and authoritative data source for many academic disciplines, it may also lead to omitting articles from other sources. Second, we only analyzed the literature in which the language was English, which may have missed some other excellent articles. In addition, there may be selection bias in the screening process due to the manual exclusion of literature that is not relevant to the research topic, although all the authors are involved in this process.

## Author contributions

WL, YW, and YH designed this study. WL, GY, and WT retrieved and collected the data. WL, YL, and LZ analyzed the data. WL wrote the manuscript. YW and YH reviewed the manuscript. All authors contributed to the article and approved the submitted version.

## Funding

This work was supported by Three-year Action Plan for Shanghai [project number: ZY (2021-2023)-0211]; National Natural Science Foundation of China (81973730); Local Colleges Faculty Constitution of Shanghai MSTC 2022 (22010504300); and Shanghai Collaborative Innovation Center for Chronic Disease Prevention and Health Services (2021 Science and Technology 02-37).

## Conflict of interest

The authors declare that the research was conducted in the absence of any commercial or financial relationships that could be construed as a potential conflict of interest.

## Publisher's note

All claims expressed in this article are solely those of the authors and do not necessarily represent those of their affiliated organizations, or those of the publisher, the editors and the reviewers. Any product that may be evaluated in this article, or claim that may be made by its manufacturer, is not guaranteed or endorsed by the publisher.
