# Supplementary material for: Bibliometric and visual analysis of nocturnal enuresis from 1982 to 2022
Source: Front Pediatr. 2022 Aug 12;10:972751. doi: 10.3389/fped.2022.972751 (PMC9412014; doi:10.3389/fped.2022.972751)
Supplement: Supplementary file 1 [file Data_Sheet_1.pdf]

Supplementary Material

1 Supplementary Figures and Tables

1.1 Supplementary Figures

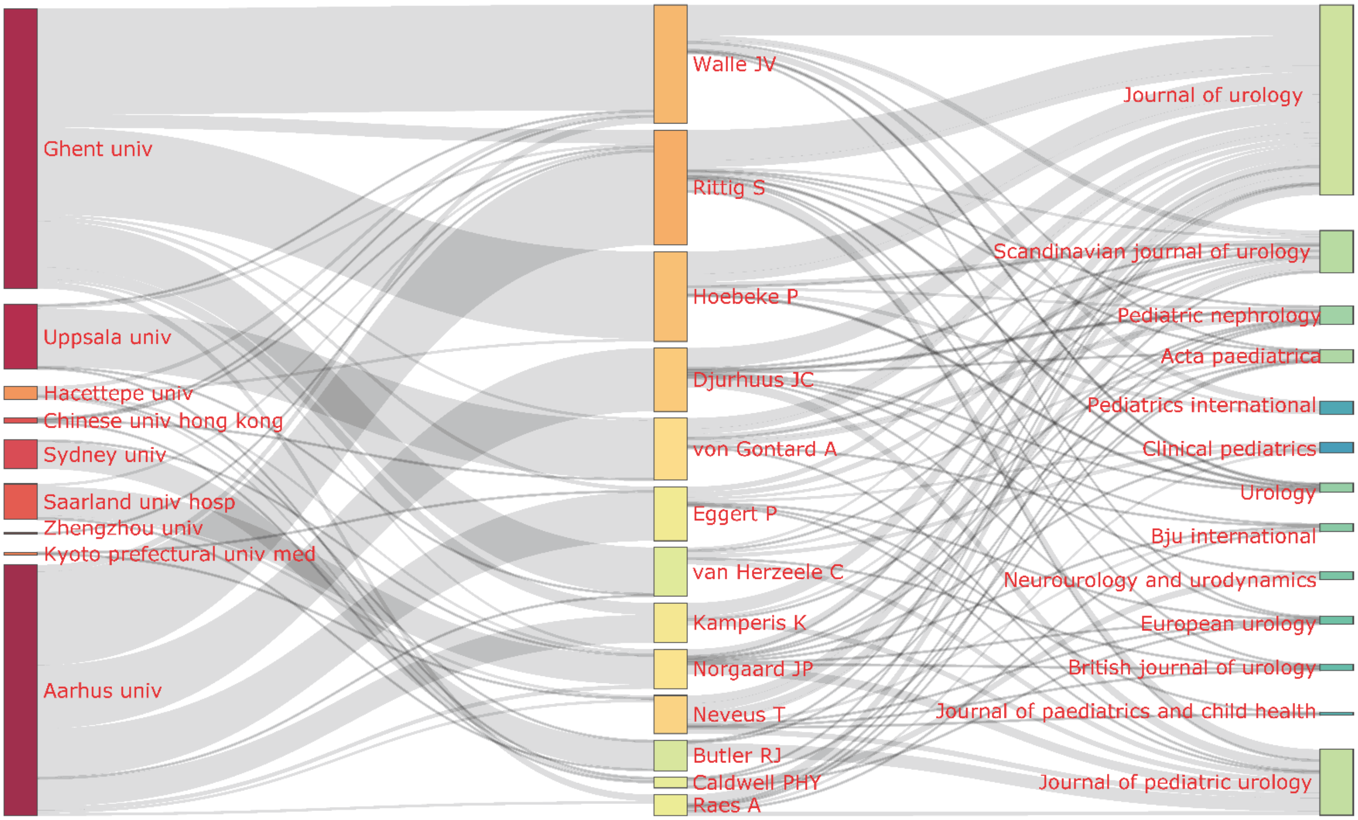

Supplementary Figure 1. Three-Fields Plot of authors, institutions, and journals. The three columns from left to right represent the organization, the author, and the journal. The thickness of the link on the left represents the proportion of articles published in the institution. The link on the right represents the proportion of articles published in the journal.

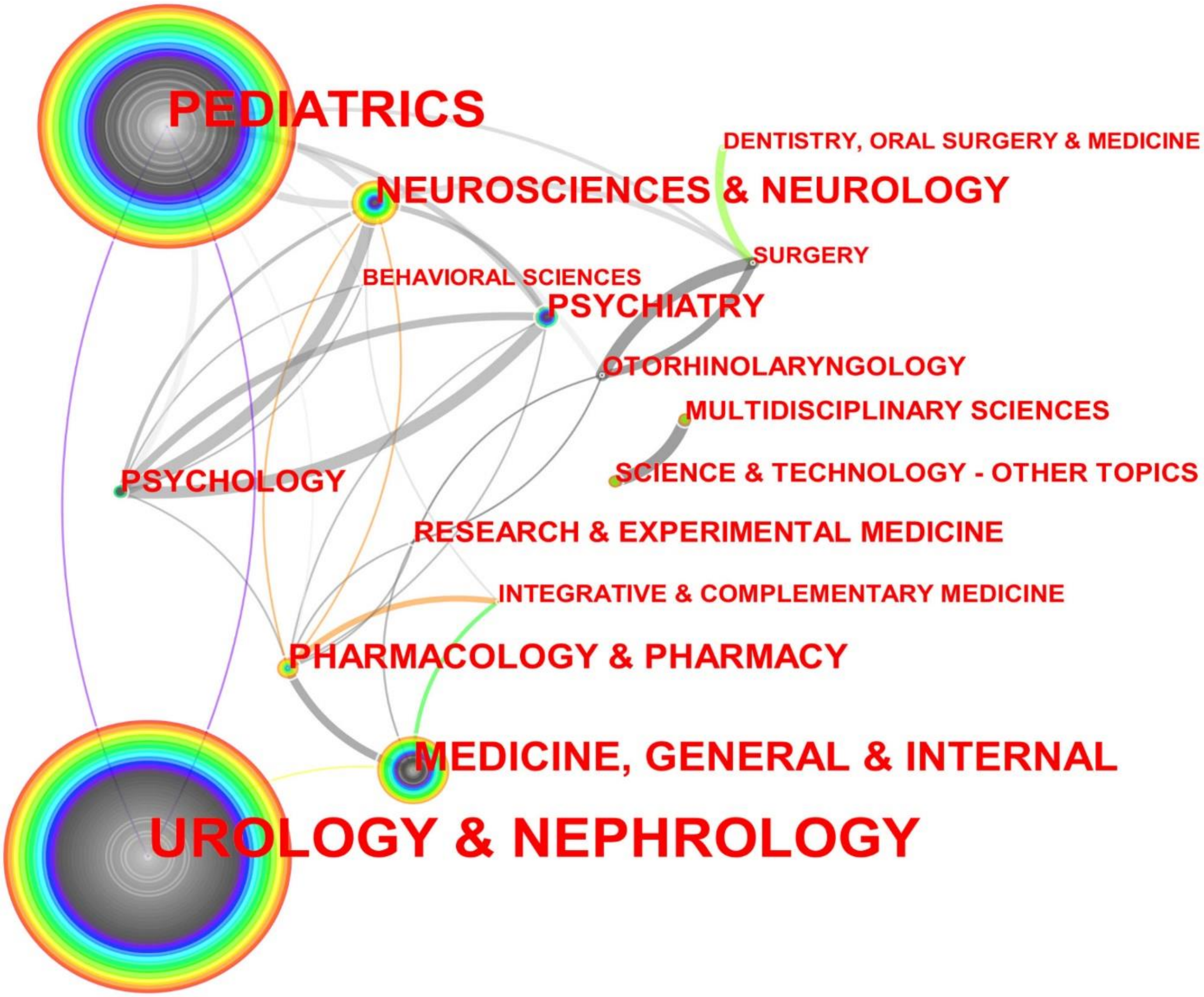

Supplementary Figure 2 Network of disciplinary on NE research. Each circle represents a subject. The size of the circle is positively related to the frequency of the subject, and the thickness of the line is positively correlated with the degree of association.

1.2 Supplementary Tables

Supplementary Table 1 Number of publications in first-author units at the top 10 institutions in NE in terms of number of publications from 1982 to 2022

Rank	First-author Affiliation	Papers	Citations	Average Citation	Countries
1	Aarhus University	45	631	14.02	Belgium
2	Ghent University	42	527	12.55	Denmark
3	Uppsala University	33	619	18.76	Sweden
4	Saarland University	15	185	12.33	Germany
5	Chinese University of Hong Kong	13	281	21.62	China
6	China Medical University	10	69	6.90	China
6	Sao Paulo University	10	31	3.10	Germany
7	Juntendo University	9	29	3.22	Japan
8	Sydney University	8	79	9.88	Australia
9	Children's Hosp Westmead	6	55	9.17	Australia
9	Zhengzhou University	6	42	7.00	China
9	Shanghai Jiao Tong University	6	12	2.00	China

Supplementary Table 2 The top 20 keywords related to NE from 1991 to 2021

Rank	Keywords	Count	Rank	Keywords	Count
1	nocturnal enuresis	550	11	alarm	95
2	children	404	12	primary nocturnal enuresis	93
3	bladder	261	13	standardization	83
4	desmopressin	205	14	adhd	65
5	incontinence	167	15	monosymptomatic nocturnal enuresis	65
6	prevalence	139	16	efficacy	62
7	adolescent	135	17	epidemiology	51
8	sleep	131	18	therapy	42
9	vasopressin	131	19	quality of life	41
10	management	96	20	terminology	35

Supplementary Table 3 The top 10 most highly cited documents on NE

Rank	First author	Title and Publication year	Citations	DOI	Journals
1	Yeung, C. K.	Differences in characteristics of nocturnal enuresis between children and adolescents: a critical appraisal from a large epidemiological study. (2004)	31	10.1111/j.1464-410x.2006.06074.x	BJU Int
2	Rittig, S.	Abnormal diurnal rhythm of plasma vasopressin and urinary output in patients with enuresis. (1989)	30	10.1152/ajprenal.1989.256.4.F664	Am J Physiol
3	Monda, J. M.	Primary nocturnal enuresis - a comparison among observation, imipramine, desmopressin acetate and bed-wetting alarm systems. (1995)	30	10.1016/S0022-5347(01)67152-0	J Urol
4	Yeung, C. K.	Reduction in nocturnal functional bladder capacity is a common factor in the pathogenesis of refractory nocturnal enuresis. (2002)	28	10.1046/j.1464-410X.2002.02884.x	BJU Int
5	Yeung, C. K.	Bladder dysfunction in children with refractory monosymptomatic primary nocturnal enuresis. (1999)	28	10.1016/S0022-5347(01)68062-5	J Urol
6	Butler, R. J.	The prevalence of infrequent bedwetting and nocturnal enuresis in childhood. (2008)	24	10.1080/00365590701748054	Scand J Urol
7	Eiberg, H.	Assignment of dominant inherited nocturnal enuresis (enur1) to chromosome 13q. (1995)	24	10.1038/ng0795-354	Nat Genet
8	von Gontard, A.	Comorbidity of ADHD and incontinence in children. (2015)	24	10.1007/s00787-014-0577-0	Eur Child Adolesc Psychiatry
9	von Gontard, A.	Psychological and psychiatric issues in urinary and fecal incontinence. (2011)	21	10.1016/j.juro.2010.11.051	J Urol
10	Arnell, H.	The genetics of primary nocturnal enuresis: Inheritance and suggestion of a second major gene on chromosome 12q. (1997)	21	10.1136/jmg.34.5.360	J Med Genet

Supplementary Table 4 A list of Randomized Controlled Trials on NE

NO.	Intervention	Duration	Patients Design	Age	Outcomes	References	Year
1	Urox® (Capsule with extractive of *Crataeva nurvala, Equisetum arvense, Lindera aggregate*)	8weeks	Urox® group: 1 capsule in the morning (< 40 kg) or 2 capsules in the morning (> 40 kg) (n=24);<br>Placebo group: Vegetarian capsule containing color-matched cellulose (n=24)	mean age of 8.61 years	Compared with the placebo group, in Urox® group 41.7% of children had improved nocturnal enuresis and significantly reduced urination urgency and underwear contamination.	Schloss J *et al*. (1)	2021
2	Adenotonsillectomy	after 7 months	Adenotonsillectomy group(n=59);<br>watchful waiting group(n=68)	5.0-9.9	The number of patients in adenotonsillectomy group decreased to 38, while there was no significant change in watchful waiting	Snow A *et al*. (2)	2021
3	Desmopressin + Propiverine	3 months	Monotherapy group: Desmopressin(0.12mg) (n=29);<br>Combination group: Desmopressin(0.12mg) + Propiverine (5mg) (n=23)	6-14	The complete remission rate of the combined treatment group was higher than that of the single treatment group (44.0% vs. 22.4%), the average functional bladder capacity increased significantly, and the risk of recurrence 6 months after the cessation of treatment was reduced	Shim M *et al*. (3)	2021
4	Solifenacin + Imipramine	3 months	Group A: Solifenacin (5-10mg) + Imipramine(25mg) (n=44);<br>Group B: Placebo(n=41)	6.5-15	Compared with placebo, the average number of wet nights in the experimental group (5.82) was significantly less than that in the placebo group (16.8), the effective rate (45.4%) was significantly higher than that in the placebo group (4.8%), and the recurrence rate (19.35%) was	Samir M *et al*. (4)	2021

					significantly lower than that in the placebo group (55.5%).		
5	Rapid maxillary expansion	14 days to 6 months	Intervention Group: Rapid Maxillary Expansion (RME)(n=18);<br>Placebo Group: RME with Sham-appliance in the former 14days, then it was consistent with the intervention group(n=18)	mean age of 10.2 years	The number of wet nights (mean-2.2) decreased more in the short-term (14 days) intervention group than in the placebo group (mean-0.6). After 6 months, the number of wet nights in the whole group decreased significantly, from 11.9 to 8.5, with an average difference of - 3.2	Ring IJ *et al*. (5)	2020
6	Alarm therapy	8-16 weeks	Experimental group (body-worn alarm, n=41);<br>Control group (bell-and-pad alarm, n=45)	6-16	Dryness was achieved in 18 children (43.9%) in the experimental group，29 children (64.4%) in the control group.	Peck B *et al*. (6)	2020
7	Checklist method	8 weeks	Group A: behavioral therapy (n = 27);<br>Group B: behavioral therapy with a written checklist (n = 26);<br>Group C: behavioral therapy with desmopressin(0.12mg) (n = 26)	6-15	Checklist method enhances treatment compliance and response of behavioral therapy	Hascicek AM *et al*. (7)	2019
8	Reboxetine	12 weeks	Group A: Reboxetine(4mg) + Placebo;<br>Group B: Reboxetine (4 mg) +Desmopressin(0.4mg);<br>Group C: Double placebo	mean age of 10 years	The reduction rate of wet night treated with riboxetine alone or in combination with desmopressin was better than that treated with placebo.And	Lundmark E *et al*. (8)	2016

					reboxetine will have mild and reversible side effects		
9	Electrical stimulation	1 year	Interferential group: standard urotherapy + 15 sessions of interferential electrical stimulation for 20 min twice per week (n=27); Control group: standard urotherapy (n=27)	6-14	15/27 (55.5 %) and 6/27 (22 %) of children in the interferential and control groups responded to treatment.	Kajbafzadeh AM *et al*. (9)	201 5
10	Repetitive sacral root magnetic stimulation (rSMS)	1 month	Real group: (rSMS; 15 Hz with a total of 1500 pulses/session for 10 sessions n = 22); Sham group (n = 19)	mean age of 13.6 years	Compared with sham group (6.5 ± 1.3 to 5.2 ± 3.2), the frequency of wet night of real group (5.7 ± 2.2 to 1 ± 1.9) decreased significantly after 1 month.	Khedr EM *et al*. (10)	201 5
11	Desmopressin + Acupuncture	3 months	Group A: Acupuncture(n=62); Group B: Desmopressin(n=62); Group C: Acupuncture + Desmopressin(n=62)	mean age of 15.7 years	The cure rate of group C was statistically significantly higher than that of group A, B and C, 33, 35 and 46 patients respectively. The bladder volume of patients treated with acupuncture and moxibustion increased significantly (groups A and C).	Moursy EES *et al*. (11)	201 4
12	Desmopressin + Oxybutynin	4 weeks	Group A: Desmopressin(0.24mg) + Oxybutynin(5mg) (n=61); Group B: Desmopressin(0.24mg) + Placebo(5mg) (n=59)	6-13	Complete and partial remission rates (45%) were higher in the oxybunin group than in the placebo group (17%)	Montaldo P *et al*. (12)	201 2

13	Ibuprofen	14 days	Group A: Ibuprofen (12.5 mg/kg) (n=78); Group B: Pseudoephedrine (15 mg/30 mg) (n=76); Group C: Ibuprofen + Pseudoephedrine(N=82); Group D: Placebo(n=82)	mean age of 8.0 years	Compared with placebo, the number of wet nights with ibuprofen alone and in combination with ibuprofen and pseudoephedrine decreased by an average of 26% and 28%. But pseudoephedrine will not enhance or weaken the efficacy of ibuprofen.	Gelotte CK *et al*. (13)	2009
14	Behavioral therapy	6 months to 3 years	Group A: Lifting with password(n=140); Group B: Lifting without password(n=143); Group C: Reward (star chart) (n=143); Group D: Control (144).	mean age of 5 years	After six months, only the group B of dry children was significantly higher (37%) than the control group (21%). After three years, the dry children in group A and group B (78%) were both significantly higher than those in the control group (69%).	van Dommelen P *et al*. (14)	2009
15	Desmopressin + Tolterodine	1 month	Desmopressin (0.6mg) + Tolterodine (0.4mg) (n=18); Desmopressin (0.6mg) + Placebo(0.4mg) (n=16)	mean age of 10.5 years	Compared with placebo, the combined treatment group with tolterodine significantly reduced the average number of wet nights and showed higher complete and partial response rates (44% success).	Austin PF *et al*. (15)	2008
16	Holding Exercises	12 weeks	Group A: holding exercises with placebo (n=29); Group B: holding exercises with oxybutynin (0.4 ml/kg) (n=30); Group C: placebo (n=30); Group D: oxybutynin (0.4 ml/kg) (n=30);	5.9-12.7	In children with monosymptomatic nocturnal enuresis, holding exercise can significantly increase the maximum urine output, but oxybutynin alone can't increase the maximum urine output	Van Hoeck KJ *et al*. (16)	2007

			Group E (controls): alarm treatment (n=30).				
17	Desmopressin	6 weeks	First three weeks, Group A: desmopressin (melt) (n=109); Group B: desmopressin tablet(n=109); After three weeks, Group A: desmopressin tablet(n=109); Group B: desmopressin (melt) (n=109)	5-15	55.7% preferred the melt formulation whose compliance (94.5%) was higher than that of tablets (88.9%) and No water intake is required. At the same time, compared with tablets, it still maintains a similar level of efficacy and safety at lower doses.	Lottmann H *et al*. (17)	2007

References

1. Schloss J, Ryan K, Steel A. A Randomised, Double-Blind, Placebo-Controlled Clinical Trial Found That a Novel Herbal Formula Urox (R) (Bedtime Buddy (R)) Assisted Children for the Treatment of Nocturnal Enuresis. *Phytomedicine* (2021) 93:153783. doi: 10.1016/j.phymed.2021.153783.

2. Snow A, Vazifedan T, Baldassari CM. Evaluation of Nocturnal Enuresis after Adenotonsillectomy in Children with Obstructive Sleep Apnea: A Secondary Analysis of a Randomized Clinical Trial. *JAMA Otolaryngol* (2021) 147(10):887-92. Epub 2021/09/10. doi: 10.1001/jamaoto.2021.2303.

3. Shim M, Bang WJ, Oh CY, Kang MJ, Cho JS. Effect of Desmopressin Lyophilisate (Melt) Plus Anticholinergics Combination on Functional Bladder Capacity and Therapeutic Outcome as the First-Line Treatment for Primary Monosymptomatic Nocturnal Enuresis: A Randomized Clinical Trial. *Investig Clin Urol* (2021) 62(3):331-9. doi: 10.4111/icu.20200303.

4. Samir M, Mahmoud MA, Elawady H. Can the Combined Treatment of Solifenacin and Imipramine Has a Role in Desmopressin Refractory Monosymptomatic Nocturnal Enuresis? A Prospective Double-Blind Randomized Placebo-Controlled Study. *Urologia* (2021) 88(4):369-73. doi: 10.1177/0391560321993587.

5. Ring IJ, Neveus T, Markstrom A, Magnuson A, Bazargani F. Rapid Maxillary Expansion in Children with Nocturnal Enuresis: A Randomized Placebo-Controlled Trial. *Angle Orthod* (2020) 90(1):31-8. doi: 10.2319/031819-219.1.

6. Peck B, Peck B, Harvey J, Green A, Svedas K, Whitaker S, et al. Body-Worn Versus Bell-and-Pad Alarm Device for the Management of Monosymptomatic Nocturnal Enuresis in Children a Randomized Controlled Trial. *J Wound Ostomy Cont Nurs* (2020) 47(5):507-12. doi: 10.1097/WON.0000000000000678.

7. Hascicek AM, Kilinc MF, Yildiz Y, Yuceturk CN, Doluoglu OG. A New Checklist Method Enhances Treatment Compliance and Response of Behavioural Therapy for Primary Monosymptomatic Nocturnal Enuresis: A Prospective Randomised Controlled Trial. *World J Urol* (2019) 37(6):1181-7. Epub 2018/09/08. doi: 10.1007/s00345-018-2478-1.

8. Lundmark E, Stenberg A, Hagglof B, Neveus T. Reboxetine in Therapy-Resistant Enuresis: A Randomized Placebo-Controlled Study. *J Pediatr Urol* (2016) 12(6):397.e1-.e5. doi: 10.1016/j.jpurol.2016.04.048.

9. Kajbafzadeh AM, Sharifi-Rad L, Mozafarpour S, Ladi-Seyedian SS. Efficacy of Transcutaneous Interferential Electrical Stimulation in Treatment of Children with Primary Nocturnal Enuresis: A Randomized Clinical Trial. *Pediatr Nephrol* (2015) 30(7):1139-45. doi: 10.1007/s00467-014-3039-5.

10. Khedr EM, Elbeh KA, Baky AA, Abo-Elfetoh N, El-Hammady DH, Korashy F. A Double-Blind Randomized Clinical Trial on the Efficacy of Magnetic Sacral Root Stimulation for the Treatment of Monosymptomatic Nocturnal Enuresis. *Restor Neurol Neurosci* (2015) 33(4):435-45. doi: 10.3233/RNN-150507.

11. Moursy EES, Kamel NF, Kaseem AF. Combined Laser Acupuncture and Desmopressin for Treating Resistant Cases of Monosymptomatic Nocturnal Enuresis: A Randomized Comparative Study. *Scand J Urol* (2014) 48(6):559-64. doi: 10.3109/21681805.2014.922609.

12. Montaldo P, Tafuro L, Rea M, Narciso V, Iossa AC, Del Gado R. Desmopressin and Oxybutynin in Monosymptomatic Nocturnal Enuresis: A Randomized, Double-Blind, Placebo-Controlled Trial and an Assessment of Predictive Factors. *BJU Int* (2012) 110(8B):E381-E6. doi: 10.1111/j.1464-410X.2011.10918.x.

13. Gelotte CK, Prior MJ, Gu J. A Randomized, Placebo-Controlled, Exploratory Trial of Ibuprofen and Pseudoephedrine in the Treatment of Primary Nocturnal Enuresis in Children. *Clin Pediatr (Phila)* (2009) 48(4):410-9. doi: 10.1177/0009922809332593.

14. van Dommelen P, Kamphuis M, van Leerdam FJM, de Wilde JA, Rijpstra A, Campagne AE, et al. The Short- and Long-Term Effects of Simple Behavioral Interventions for Nocturnal Enuresis in Young Children: A Randomized Controlled Trial. *J Pediatr* (2009) 154(5):662-6. doi: 10.1016/j.jpeds.2008.12.001.

15. Austin PF, Ferguson G, Yan Y, Campigotto MJ, Royer ME, Coplen DE. Combination Therapy with Desmopressin and an Anticholinergic Medication for Nonresponders to Desmopressin for Monosymptomatic Nocturnal Enuresis: A Randomized, Double-Blind, Placebo-Controlled Trial. *Pediatrics* (2008) 122(5):1027-32. doi: 10.1542/peds.2007-3691.

16. Van Hoeck KJ, Bael A, Van Dessel E, Van Renthergem D, Bernaerts K, Vandermaelen V, et al. Do Holding Exercises or Antimuscarinics Increase Maximum Voided Volume in Monosymptomatic Nocturnal Enuresis? A Randomized Controlled Trial in Children. *J Urol* (2007) 178(5):2132-6. doi: 10.1016/j.juro.2007.07.051.

17. Lottmann H, Froeling F, Alloussi S, El-Radhi AS, Rittig S, Riis A, et al. A Randomised Comparison of Oral Desmopressin Lyophilisate (Melt) and Tablet Formulations in Children and Adolescents with Primary Nocturnal Enuresis. *Int J Clin Pract* (2007) 61(9):1454-60. doi: 10.1111/j.1742-1241.2007.01493.x.
